# Supplementary material for: Causal Relationship between Aging and Anorexia Nervosa: A White-Matter-Microstructure-Mediated Mendelian Randomization Analysis
Source: Biomedicines. 2024 Aug 16;12(8):1874. doi: 10.3390/biomedicines12081874 (PMC11351342; doi:10.3390/biomedicines12081874)
Supplement: Supplementary file 1 [file biomedicines-12-01874-s001.zip › Supplementary Figure/Figure S4/Singe SNP plot of MR analysis for TL to ubm-b-1783.pdf]

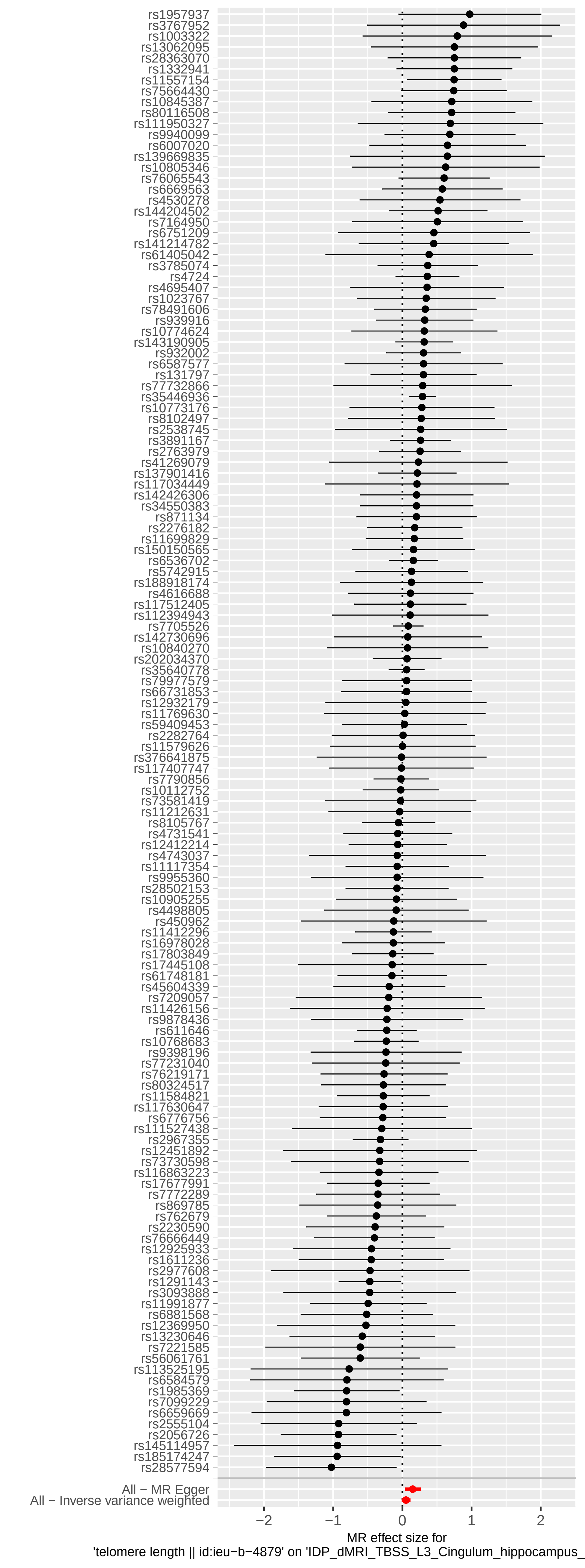

rs1957937
rs3767952
rs1003322
rs13062095
rs28363070
rs1332941
rs11557154
rs75664430
rs10845387
rs80116508
rs111950327
rs9940099
rs6007020
rs139669835
rs10805346
rs76065543
rs6669563
rs4530278
rs144204502
rs7164950
rs6751209
rs141214782
rs61405042
rs3785074
rs4724
rs4695407
rs1023767
rs78491606
rs939916
rs10774624
rs143190905
rs932002
rs6587577
rs131797
rs77732866
rs35446936
rs10773176
rs8102497
rs2538745
rs3891167
rs2763979
rs41269079
rs137901416
rs117034449
rs142426306
rs34550383
rs871134
rs2276182
rs11699829
rs150150565
rs6536702
rs5742915
rs188918174
rs4616688
rs117512405
rs112394943
rs7705526
rs142730696
rs10840270
rs202034370
rs35640778
rs79977579
rs66731853
rs12932179
rs11769630
rs59409453
rs2282764
rs11579626
rs376641875
rs117407747
rs7790856
rs10112752
rs73581419
rs11212631
rs8105767
rs4731541
rs12412214
rs4743037
rs11117354
rs9955360
rs28502153
rs10905255
rs4498805
rs450962
rs11412296
rs16978028
rs17803849
rs17445108
rs61748181
rs45604339
rs7209057
rs11426156
rs9878436
rs611646
rs10768683
rs9398196
rs77231040
rs76219171
rs80324517
rs11584821
rs117630647
rs6776756
rs111527438
rs2967355
rs12451892
rs73730598
rs116863223
rs17677991
rs7772289
rs869785
rs762679
rs2230590
rs76666449
rs12925933
rs1611236
rs2977608
rs1291143
rs3093888
rs11991877
rs6881568
rs12369950
rs13230646
rs7221585
rs56061761
rs113525195
rs6584579
rs1985369
rs7099229
rs6659669
rs2555104
rs2056726
rs145114957
rs185174247
rs28577594
All − MR Egger
All − Inverse variance weighted
−2
−1
0
1
2
MR effect size for
'telomere length || id:ieu−b−4879' on 'IDP_dMRI_TBSS_L3_Cingulum_hippocampus_
